# Marital Histories and Late-Life Economic Security: Do Social Security Benefits Rules Perpetuate Disparities?

**DOI:** 10.1093/geroni/igab046.928

**Published:** 2021-12-17

**Authors:** Deborah Carr

**Affiliations:** Boston University, Boston, Massachusetts, United States

## Abstract

Disparities in late-life economic security persist along the lines of gender, marital status, race, and educational attainment. We propose that these disparities are partly due to the fact that Social Security benefits are structured such that never-married, divorced, and cohabiting persons, those who were widowed prematurely, or were in a dual-earner couple face benefit penalties. Drawing on data from the Wisconsin Longitudinal Study (WLS), a study that has followed men and women from age 18 (in 1957) through age 72 (in 2011), we examine disparities in Social Security earnings and poverty risk on the basis of gender and marital histories. Our results reveal a large disadvantage for divorced and never-married persons (relative to their married counterparts), with women and those divorced two or more times experiencing the largest toll. We discuss the implications of our results for revamping Social Security to better meet the needs of 21st century families.

